# An unusual case of chronic mesenteric ischemia: Case report

**DOI:** 10.1016/j.radcr.2023.05.058

**Published:** 2023-07-12

**Authors:** Qusay Abdoh, Mohammad Alnees, Tayseer Sabooh, Zaid Sowaity

**Affiliations:** a Department of Gastroenterology, An-Najah National University Hospital, Nablus, Palestine; b Department of Medicine, Faculty of Medicine and Health Sciences, An-Najah National University, Nablus, Palestine; c Faculty of Medicine, Al Quds University, Jerusalem, Palestine

**Keywords:** Unusual, Chronic, Mesenteric, Ischemia, Case report, Abdominal pain

## Abstract

Chronic mesenteric ischemia (CMI) is a rare cause of abdominal pain with risk factors as Diabetes, Hypertension, smoking and age above 65-year-old age. A 55 -year-old man, a heavy smoker, with no other risk factor for chronic mesenteric ischemia, presented with a recurrent episodes of abdominal pain. Many differential diagnoses were excluded, CT angiography was showed Inferior Mesenteric artery (IMA) and superior Mesenteric artery (SMA) stenosis, then the Intervention was done successful. Gastric ulcers that are resistant to treatment, H. pylori negative and with no history of non-steroidal anti-inflammatory drugs (NSAID) use should be investigated for a possible ischemic.

## Introduction

Chronic mesenteric ischemia is a rare, but potentially fatal [1]. CMI is an abdominal artery-occlusive disease that causes abdominal symptoms due to poor blood supply to the gastrointestinal tract [2]. In symptomatic patients’ treatment is required because of a greatest risk of bowel infarction [3]. The etiology of the disease is stenosis or occlusion of independent or multiple mesenteric arteries such as the superior mesenteric artery (SMA), celiac artery (CA), and inferior mesenteric artery (IMA) [2]. Diagnosis is frequently delayed due to nonspecific manifestations and rare incidence, accounting for less than one in 1000 hospital admissions for abdominal pain [4].

## Case presentation

**Fig. 1:**
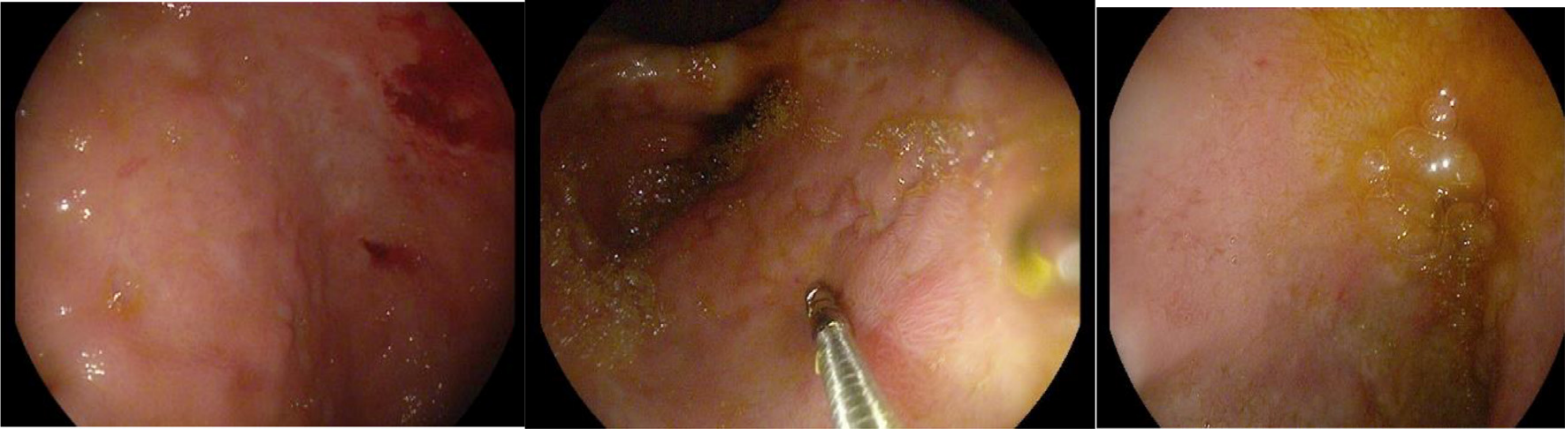
Gastroscopy revealed multiple antral healed ulcers and aphthous ulceration and a biopsy showed mild chronic inflammation with no metaplasia.

A 55-year-old man, who is a heavy smoker, he didn't have remarkable past medical history for example diabetes, hypertension, hyperlipidemia, peripheral artery disease, and coronary artery disease. And past surgical history with calculous cholecystectomy. He was admitted to the hospital with a 6-month history of abdominal pain. His complaint began as epigastric pain that worsened with fatty meals and improved with fasting states, accompanied by significant weight loss, and no other symptoms such as vomiting, melena, joint pain, fever, or a skin rash. A gastroscopy revealed multiple antral healed ulcers and aphthous ulceration with no Helicobacter pylori infection; and a biopsy showed mild chronic inflammation with no metaplasia (Fig. 1).

**Fig. 2:**
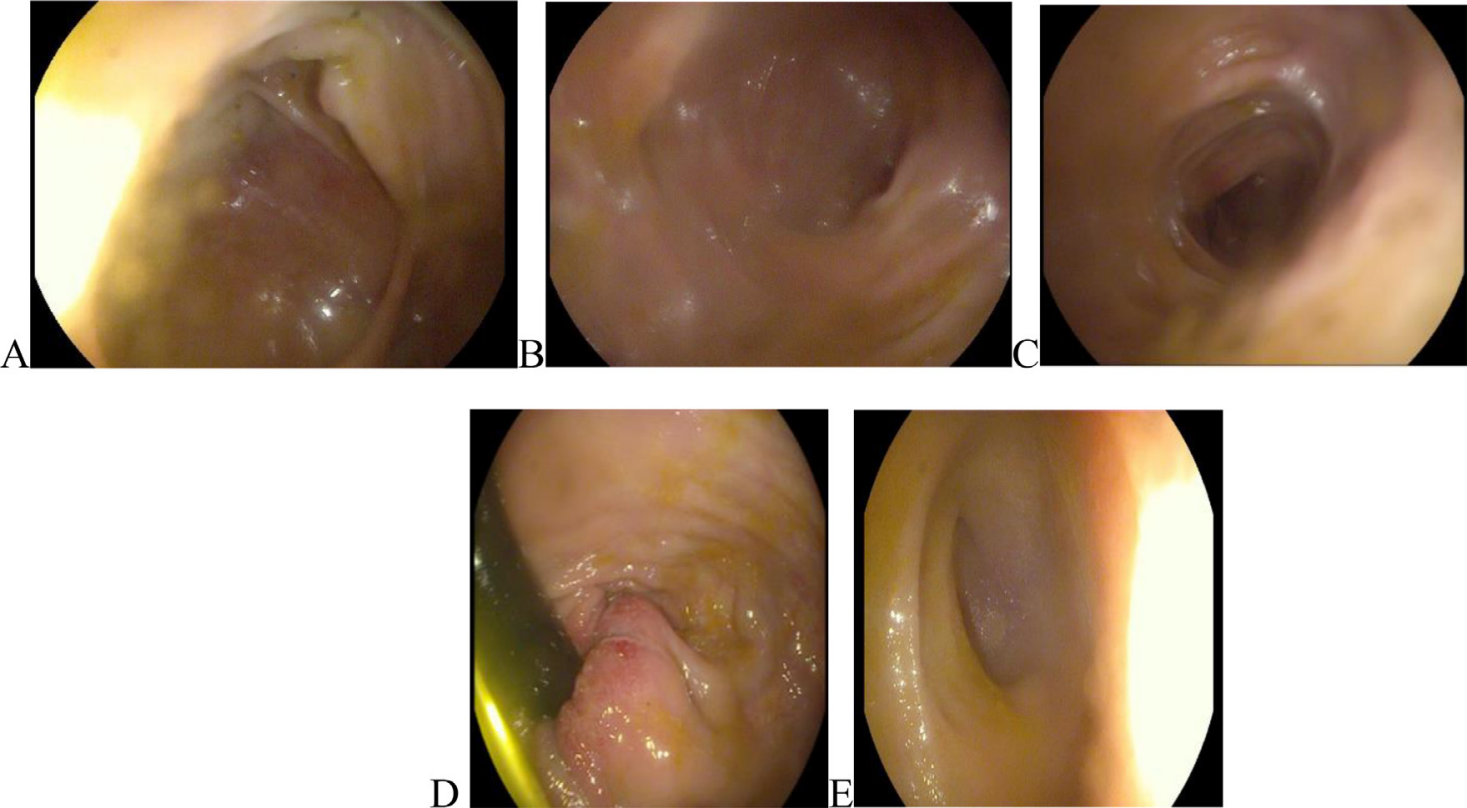
colonoscopy with, (A, B, C, E) grossly normal terminal ileum and colonic mucosa, (D) active hemorrhoids.

He was discharged on proton pump inhibitors (PPI) with a recommendation for colonoscopy. The patient's condition didn't improve on PPI. So, the patient was referred to the hospital for a colonoscopy. Result showed grossly normal terminal ileum and colonic mucosa and with active hemorrhoids (Fig. 2). He was discharged on laxatives.

An IV and oral contrast CT scan of the abdomen and pelvis were performed twice with normal results. Patient is still suffering, his abdominal pain became generalized mainly in the lower abdominal area associated with watery diarrhea, abdominal bloating, and 18 kg weight loss in the last 4 months.

**Fig. 3:**
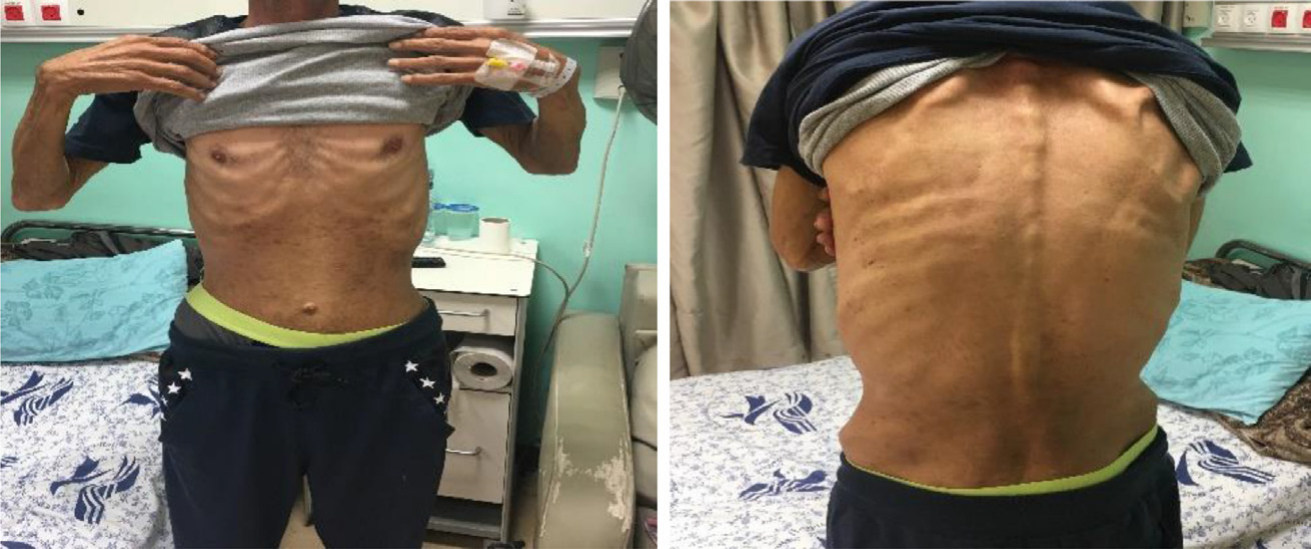
Significant weight loss (Cachectic) in the abdomen and back.

The patient was cachexic (BMI 18, not pale or jaundiced) (Fig. 3). His vital signs were blood pressure (BP) 125/70 mm Hg, heart rate (HR) 85 bpm, temperature 37°C, and oxygen saturation (SAT O2) 99%. Examination revealed a scaphoid abdomen, scars from previous laparoscopic cholecystectomy, diffused tenderness primarily over the lower abdomen and epigastric area. Extremities: +1 lower limb edema bilaterally. Head, neck, chest, and heart were unremarkable.

Labs on the admission were described in Table 1, hepatitis markers and Chest X-ray were unremarkable.

**Table 1:** The patient's labs on admission.

Labs	Result	Reference range
WBC in CBC	14.9 10^3^/µL	(4.6-11) 10^3^/µL
HGB in CBC	9.6 g/dL	13.5-17 g/dL
PLT in CBC	250 10^3^/µL	(150-450) 10^3^/µL
International Normalized Ratio (INR)	1.1	(0.9-1.1)
Aspartate Aminotransferase (AST)	27 U/L	(0-50) U/L
Alanine Transaminase (ALT)	49 U/L	(0-41) U/L
Bilirubin, Total	0.9 mg/dL	(0-1.2) mg/dL
Bilirubin, Direct	0.5 mg/dL	(0-0.3) mg/dL
Albumin	2.9 g/dL	(3.97-4.94) g/dL
Amylase	58 U/L	(28-100) U/L
Lipase	81 U/L	(13-60) U/L
Creatinine, Serum	0.5 mg/dL	(0.7-1.2) mg/dL
Blood Urea Nitrogen (BUN)	11 mg/dL	(8-23) mg/dL
Potassium, Serum	3.2 mmol/L	(3.5-5.3) mmol/L
Sodium, Serum	137 mmol/L	(135-145) mmol/L
Erythrocyte sedimentation rate (ESR)	30 mm/first hour	(<15) mm/first hour

Intra-abdominal malignancy (especially small bowel malignancies, pancreatic cancer), chronic pancreatitis, inflammatory bowel disease, chronic mesenteric ischemia and celiac disease were the differential diagnoses.

Celiac disease was ruled out when anti-tissue transglutaminase IgA was negative (NL IgA).

CT Scan Revision was done and showed hepatomegaly 18 cm, no signs of cirrhosis, no signs of Chronic Pancreatitis or Pancreatic Malignancies, no masses, no significant lymph nodes and Fecal Elestase-1 was negative.

**Fig. 4:**
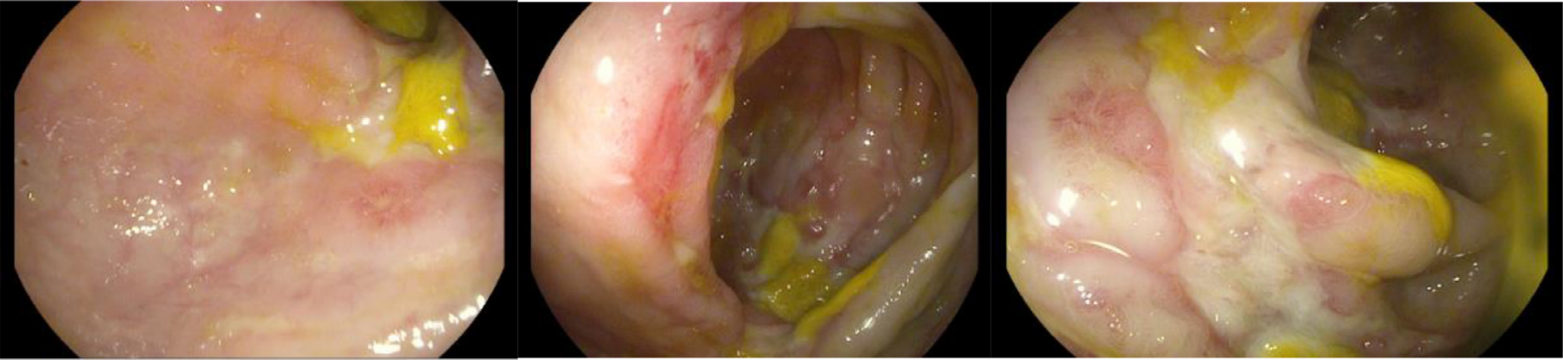
colonoscopy with congested and hyperemic colonic mucosa with deep and diffuse ulceration on the RT side of the colon.

After 6 months of abdominal pain, he underwent another colonoscopy that showed congested and hyperemic colonic mucosa with deep and diffuse ulceration involving the RT side of the colon, mainly the ileocecal valve, and sparing the cecum transverse (Fig. 4). The LT side of the colon was normal, Rectum had a large hemorrhoid, rectal varices, fecal calprotectin: 160 µg/mg (normal range <50 µg/mg). The patient was started on ciprofloxacin and metronidazole. Inflammatory Bowel Disease was ruled out by colonic biopsy.

Then CT angiography was done that showed a significant attenuation of the celiac trunk and SMA, IMA not-visualized, hypodensity in the spleen and its hilum that suggestive of ischemic insult, and there was a significant wall thinking of the rectum.

MRE revealed small bowel thickening at the ileocecal junction, but no other abnormalities.

A vascular surgeon and interventional radiologist were consulted on the plan for angioplasty versus bypass surgery.

**Fig. 5:**
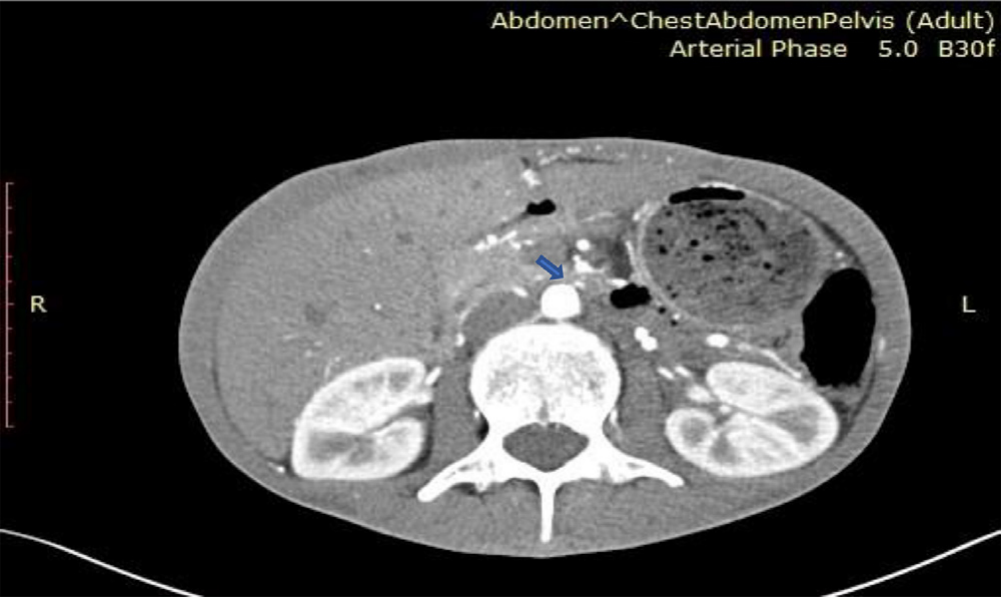
IMA stenosis on the axial view of CT Angiography with arterial phase.

**Fig. 6:**
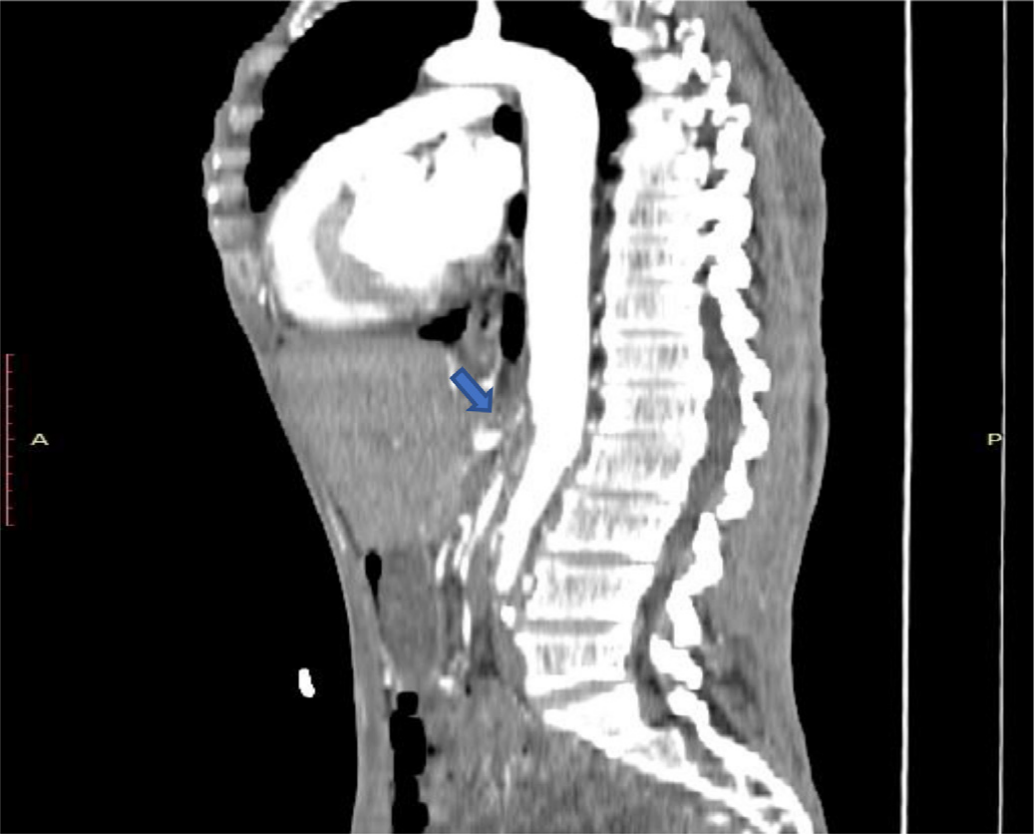
SMA total occlusion on the sagittal view of CT Angiography with arterial phase.

**Fig. 7:**
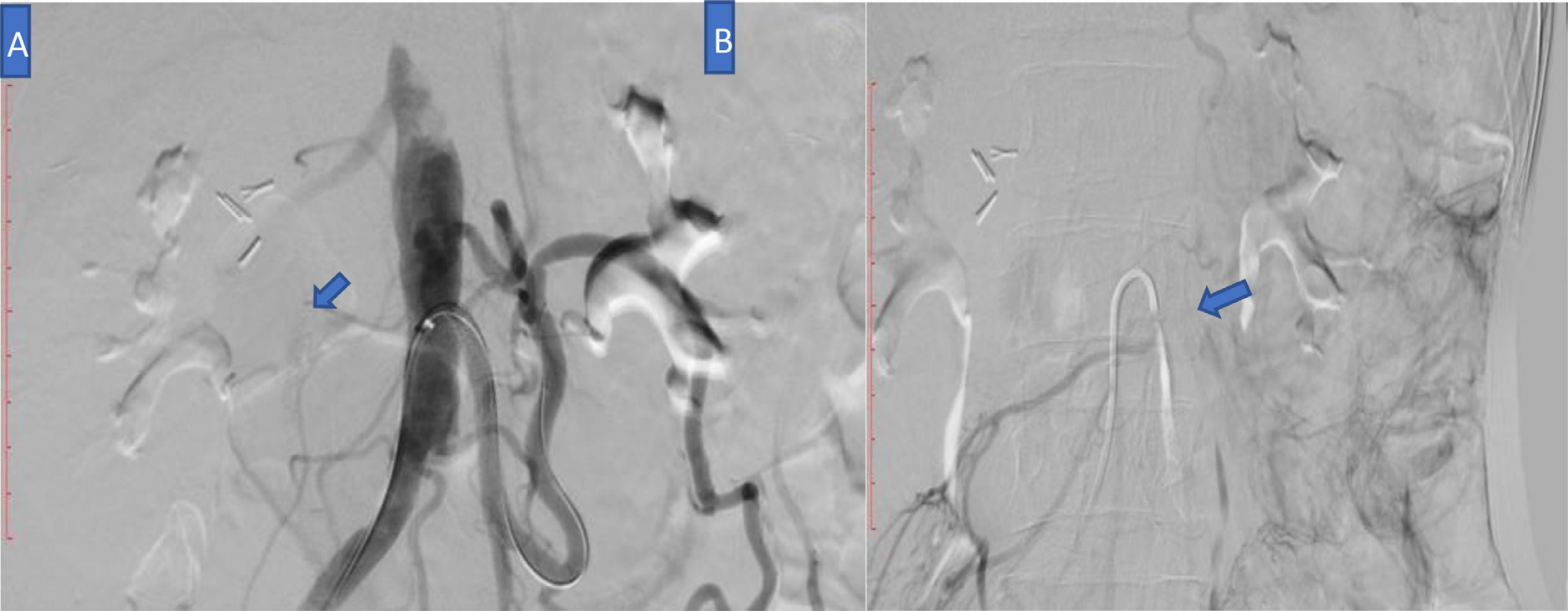
(A) IMA stenosis on the conventional radiography (B) Percutaneous transluminal angioplasty (PTA) to IMA and balloon dilatation.

After one week of admission angioplasty revealed that Inferior mesenteric Artery (IMA) had ostial severe stenosis and mid-third significant stenosis (Fig. 5). Superior Mesenteric Artery (SMA) had chronic total occlusion from the ostium with collateral from IMA and intercostal artery (Fig. 6). Intervention was successful, from Percutaneous transluminal angioplasty (PTA) to IMA and balloon dilatation (Fig. 7).

Patient was observed for two days, there were no complications, his abdominal pain was completely relieved, he tolerated diet without complications, his diarrhea subsided and he was then discharged home in a good condition on Dual Antiplatelet Therapy (DAPT), statins, antiheart failure medication and antibiotics.

## Discussion

The patient was an old smoker who presented to our hospital with postprandial epigastric pain. One of the differential diagnoses was gastric ulcer, so a gastroscopy was done, and a biopsy was taken that excluded H. pylori infection. The patient then started on PPIs, but there has been no improvement.

Other possible diagnoses included acute pancreatitis, celiac disease, and malignancies, all of these tests came back negative.

When epigastric pain transferred all over the abdomen, he underwent a colonoscopy that showed congested and hyperemic colonic mucosa with deep and diffuse ulceration that cross-matched with Crohn's disease, but the workup of Crohn's disease was negative.

When there wasn't any improvement in his symptoms, we turned to a CT angiography that finally showed CMI.

CMI is defined as visceral hypoperfusion involving at least two of the following: the celiac artery, the superior mesenteric artery, and the inferior mesenteric artery [1]. It is usually due to arteriosclerosis and presents with nonspecific systemic symptoms such as postprandial pain, weight loss, nausea, or diarrhea [4]. Factors that predispose patients to atherosclerosis are associated with an increased risk for chronic mesenteric ischemia. These include smoking, hypertension, diabetes mellitus and hypercholesterolemia [5].

The reason for the delay in diagnosing this condition is the large number of differential diagnoses that come to the fore. In addition, there were no risk factors for the patient (diabetes, hypertension, hyperlipidemia) other than smoking. Unlike our patient, it is more common in females and those over the age of 65 [6].

Gastric ulcers that are resistant to treatment or are H. pylori negative with no history of NSAID use should be investigated for a possible ischemic etiology, especially in patients with concomitant atherosclerotic vascular disease [7].

## Conclusion

In the diagnosis of chronic mesenteric ischemia patients, there were no risk factors for the patient (diabetes, hypertension, hyperlipidemia) except smoking. Unlike our patient, it is more common in females and those over the age of 65. this is a complicated issue for gastroenterologists worldwide. A delay in diagnosis and treatment can result in more danger over time, healthcare providers may recommend surgery more quickly in addition Mesenteric ischemia is treatable and reversible when it's caught early enough.

## Authors’ contributions

MA, TS, and ZS: wrote the manuscript; QA: diagnosis, management, and follow-up of the case. All authors read and approved the final manuscript the final manuscript.

## Ethical approval

The manuscript's conduct and publication have received approval from the university's IRB.

## Availability of data and materials

The corresponding author will provide the data sets used and/or analyzed during the current study upon reasonable request.

## Patient consent

Written informed consent for the publication of this case report was obtained from the patient.
